# Path planning and smoothing of mobile robot based on improved artificial fish swarm algorithm

**DOI:** 10.1038/s41598-021-04506-y

**Published:** 2022-01-13

**Authors:** Fei-Fei Li, Yun Du, Ke-Jin Jia

**Affiliations:** School of Electrical Engineering, Hebei University of Science and Technology, Shijiazhuang, 050018 China

**Keywords:** Electrical and electronic engineering, Computational science, Computer science

## Abstract

An algorithm that integrates the improved artificial fish swarm algorithm with continuous segmented Bézier curves is proposed, aiming at the path planning and smoothing of mobile robots. On the one hand, to overcome the low accuracy problems, more inflection points and relatively long planning paths in the traditional artificial fish swarm algorithm for path planning, feasible solutions and a range of step sizes are introduced based on Dijkstra's algorithm. To solve the problems of poor convergence and degradation that hinder the algorithm's ability to find the best in the later stage, a dynamic feedback horizon and an adaptive step size are introduced. On the other hand, to ensure that the planned paths are continuous in both orientation and curvature, the Bessel curve theory is introduced to smooth the planned paths. This is demonstrated through a simulation that shows the improved artificial fish swarm algorithm achieving 100% planning accuracy, while ensuring the shortest average path in the same grid environment. Additionally, the smoothed path is continuous in both orientation and curvature, which satisfies the kinematic characteristics of the mobile robot.

## Introduction

Path planning has become a hot topic as mobile robots are widely used in industrial, service and medical industries, among others. Path planning is an important area of research in mobile robotics, where the goal is to plan a collision-free route from a starting point to a target point. The path planning problem can also be formulated as an optimization problem subject to several constraints and performance criteria^1^ (e.g., shortest distance, feasibility of the path, whether kinematic constraints are satisfied). To date, a large number of algorithms have been available for path planning of mobile robots, such as the ant colony algorithm^2^, particle swarm algorithm^3^, A* algorithm^4^, artificial potential field algorithm^5^, genetic algorithm^6^, neural network algorithm^7^ and rapid exploration random tree (RRT)^8^. Among them, the ant colony algorithm is relatively sensitive to the initial parameters, and different parameters reduce the algorithm's searchability. Some individual ants become lost in the search process, and a large number of incomplete paths appear. The particle swarm algorithm requires a large number of samples to approximate the posterior probability of the system, and the validity and diversity of the samples cannot be guaranteed in the resampling stage, which leads to the depletion of the samples. Thus, the algorithm is computationally intensive. Planning paths is tedious for the A* algorithm, and the algorithm often generates a large number of invalid search paths. The artificial potential field method is prone to local optimality and deadlock. The genetic algorithm relies on experience for selecting relevant parameters and is prone to being "premature." The neural network is computationally intensive and requires a large number of data models for training. The fast exploration of randomized trees requires considerable memory due to too many nodes.

Based on the above problems, many people introduce new bionic algorithms for planning and design. The artificial fish swarm algorithm is one of them. An artificial fish swarm is a parallel stochastic search algorithm with advantages, such as good robustness, strong global searchability, large tolerance of parameter setting, and insensitivity to initial values^9^, which is suitable for path planning. However, there are also some shortcomings, and for the defects of the traditional artificial fish swarm algorithm, in^10^, the parameters *visual*, *step*, *δ* are adjusted by self-tuning in due time to achieve the purpose of improving the convergence accuracy. In^11^, an adaptive improved artificial fish swarm algorithm that ignores the crowding factor was proposed and applied to the path planning of mobile robots. In^12^, an improved artificial fish swarm algorithm based on the dynamic step range and field of view range was proposed to realize the transformation of the algorithm from coarse search to fine search, thus improving the convergence of the algorithm. In^13^, a logarithmic function adaptive artificial fish swarming algorithm (ALF-AFSA) was proposed, which uses a logarithmic function as the step range to improve the algorithm's search capability. In^14^, an aggregation degree factor is introduced to obtain the adaptive step range and field of view range. Additionally, a weight evaluation factor is introduced in the fish swarm behavior to solve the problem that the algorithm easily falls into a local optimum and is premature. In^15^, four behaviors of fish swarms were introduced with an adaptive strategy, variational strategy and hybrid strategy, and the step range and field of view range of the hyperbolic tangent function were added to optimize the optimal finding ability and improve the convergence speed, which was applied to solve the flow shop scheduling (FFS) problem^16^. The efficiency of the algorithm was proven by experiments. Additionally, the mobile robot driving path must satisfy the continuity of direction and curvature. In^17^, the path was smoothed by combining three uniform B splines. In^18^, a sequence of planned path points was fitted using the cubic Hermit spline method, which generates monotonic curves for all path points where curvature is discontinuous at the path points. However, many algorithms solve the smoothing problem either by splicing straight and circular segments or by using spline curves without considering the dynamical state of the mobile robot.

The above improved artificial fish swarming algorithms all successfully solve the deficiencies of AFSA in the corresponding application areas, but some of the improved algorithms cannot reach the desired expectations in the grid environment. In the grid environment, the traditional artificial fish swarming algorithm has two types of movement: 4 movement directions with 4 neighborhoods and 8 movement directions with 8 neighborhoods. In this paper, we propose an improved artificial fish swarm algorithm with 16 moving directions and 24 neighborhoods, which makes the artificial fish have more directional choices and wider moving range during swimming. To avoid the phenomenon of individual crossing the barrier in the 16-Direction 24-Neighborhood due to the relatively large step size of the artificial fish, Dijkstra's algorithm is introduced to determine part of the step size range of the 16-Direction 24-Neighborhood again. To overcome the problem that the global optimal solution and the local optimal solution interfere with each other, the feasible solution is introduced and the optimal path is found in the feasible solution, thus, improving the planning accuracy and ensuring the shortest planning path. To overcome the problem that the computational volume increases due to the introduction of Dijkstra's algorithm, the sharing mechanism is introduced, i.e., the fish population in the same grid point can share the obstacle information in the step length range. To ensure that the planned path is more consistent with the kinematic characteristics of the mobile robot, the path smoothing algorithm is designed for the generated polyline segments after the initial path points are obtained. Thus, the generated curve is a continuous function in both orientation and curvature of the path. Finally, the feasibility and effectiveness of the algorithm are verified by simulation experiments.

### Grid environment establishment

**Figure 1:**
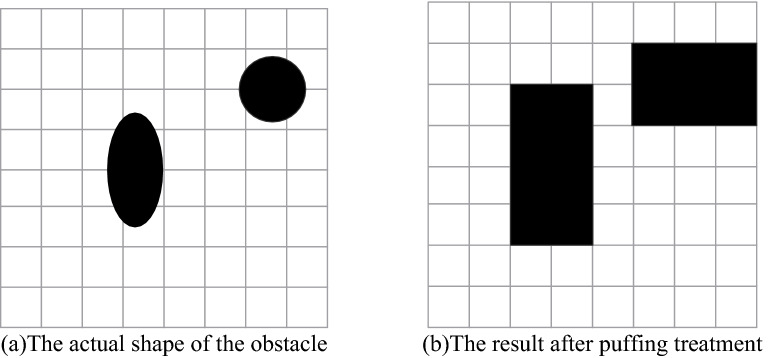
Grid map environment construction.

**Figure 2:**
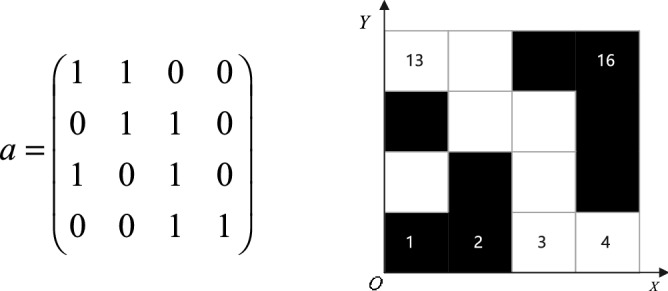
Grid environment matrix expression.

The grid graph is the most commonly used environmental modeling method in path planning. The combined algorithm of dilation and erosion of obstacles in Fig. 1a is carried out by morphology to extract features and reduce the complexity of the modeling environment to obtain Fig. 1b. This paper uses the grid method to model obstacles with 0, corresponding to black squares in the grid map, 1 representing feasible areas, and corresponding to white squares in the grid map, as shown in Fig. 2.

In the grid map, each grid has a unique coordinate location corresponding to it, and the relationship between the grid sequence number and the coordinates is as follows (1):

1 $$\left\{ {\begin{array}{*{20}l} {x = {\text{fix}}((i - 1)/N_{x} ) + 1} \hfill \\ {y = \bmod \,((i - 1),N_{y} ) + 1} \hfill \\ {N_{x} = N_{y} = {\text{size}}(a,1)} \hfill \\ \end{array} } \right.$$

In Eq. (1), (*x*, *y*) is the coordinate of the grid, fix() is the integral function, and mod() is the residual function. *N*_*x*_ and *N*_*y*_ are the maximum rows and columns of the grid map, respectively, which are equal to the maximum rows of the matrix.

In the grid map, the objective function of the optimization problem can be expressed as follows (2):

2 $$f(x,y) = \sqrt {(x_{i} - x_{goal} )^{2} + (y_{i} - y_{goal} )^{2} }$$

### The artificial fish school algorithm and the Dijkstra algorithm

**Figure 3:**
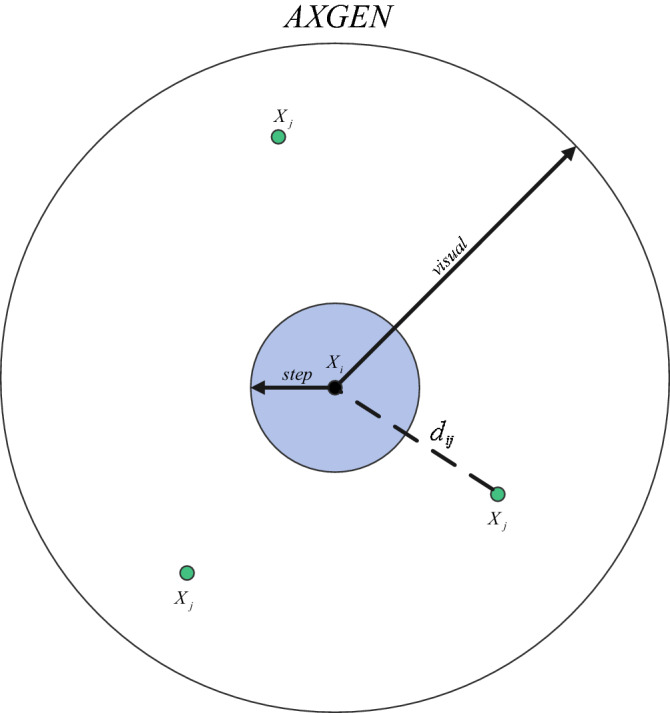
Artificial fish field of view model.

AFSA completes the process of finding the optimal solution by simulating the preying behavior, swarming behavior, following behavior, and random behavior of fish in nature. The artificial fish swarm mainly has the following parameters: the population size *N*of the fish swarm, the position $X_{i} \left( {i = 1,2,3 \ldots .,N} \right)$ of the fish swarm, the food concentration *Y*_*c*_ = *f*(*x*_*i*_) of the current position of the fish swarm, the crowding factor *δ* of the fish swarm, the number of attempts *try-number* of the fish swarm, the step size *step* of the fish swarm, the visual field range *visual* of the fish swarm, the distance between fish *X*_*i*_ and *X*_*j*_ is $d_{ij} = \parallel X_{i} - X_{j} \parallel$, and the maximum number of iterations *MAXGEN*. The model is shown in Fig. 3.

#### Prey Behavior

Prey behavior is the most important behavior of the fish swarm algorithm. Artificial fish randomly select one location *X*_*j*_ in the field of vision, and compares the food concentration of current location *X*_*i*_ and location *X*_*j*_. If the food concentration *Y*_*j*_ of the random position is less than the food concentration *Y*_*i*_ of the current position, position *X*_*j*_ will be reselected. Until the number of selections is greater than the number of attempts *try-number* of artificial fish, and no position with higher concentration than the current position is found, the random behavior is performed, and Eq. (3) is as follows:

3 $$X_{i} \left( {t + 1} \right) = X_{i} \left( t \right) + {\text{step}}*\frac{{X_{j} \left( t \right) - X_{i} \left( t \right)}}{{\parallel X_{j} \left( t \right) - X_{i} \left( t \right)\parallel }}*{\text{rand}}()$$

#### Swarm Behavior

Swarm behavior is a substantial way of survival for fish and can be used for collective foraging, as well as for the avoidance of natural enemies. There are two necessary conditions for aggregation behavior: one is close to the center of the fish group and the other is the low crowding degree of the fish group. By observing the number *n*_*f*_ and position *X*_*j*_ of all fish in the field of vision, the center position $X_{c} = \sum\nolimits_{j = 1}^{{n_{f} }} {X_{j} /} n_{f}$ is determined, and the concentration *Y*_*c*_ is calculated. If the crowding degree $n_{f} /N \le \delta$ of the current fish population and the concentration *Y*_*c*_ of the center position is greater than *Y*_*i*_, indicating that the center position *X*_*c*_ is better and not crowded, clustering behavior can be performed; otherwise, foraging behavior can be performed. Equation (4) is as follows:

4 $$X_{i} \left( {t + 1} \right) = X_{i} \left( t \right) + {\text{step}}*\frac{{X_{c} - X_{i} \left( t \right)}}{{\parallel X_{c} - X_{i} \left( t \right)\parallel }}*{\text{rand}}().$$

#### Follow Behavior

Artificial fish observe the fish within the field of vision, determine the optimal food concentration *Y*_*j*_ and its position *X*_*j*_, and are close to this position. If *Y*_*j*_ is greater than the concentration *Y*_*i*_ of the current position and the density $n_{f} /N \le \delta$ of the current fish, this shows that the better position of *X*_*j*_ can perform the pursuit behavior; otherwise it can perform the foraging behavior. Equation (5) is as follows:

5 $$X_{i} \left( {t + 1} \right) = X_{i} \left( t \right) + {\text{step}}*\frac{{X_{j} \left( t \right) - X_{i} \left( t \right)}}{{\parallel X_{j} \left( t \right) - X_{i} \left( t \right)\parallel }}*{\text{rand}}().$$

#### Random Behavior

Random behavior is a kind of default behavior for the fish swarm. In the visual field of artificial fish, the random selection of a direction to move can prevent fish from lowing efficiency and local optimum, which is more conducive to the global optimum. The formula is expressed as follows:

6 $$X_{i} \left( {t + 1} \right) = X_{i} \left( t \right) + {\text{visual}}*{\text{rand}}().$$

### Dijkstra algorithm

**Figure 4:**
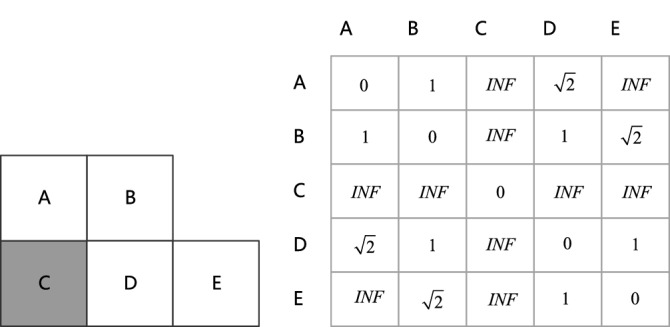
Dijkstra algorithm grid environment and its adjacency matrix.

The core idea of the Dijkstra algorithm is to take the initial point as the center point, and continuously iteratively expand to the outer layer until the target point is found. Figure 4 shows the grid map of the Dijkstra algorithm and the expression form of its adjacency matrix, and *INF* represents the unreachable.

### Improved artificial fish swarm algorithm

In the traditional AFSA, there is a problem where the global optimal solutions and the local optimal solutions interfere with each other. To avoid the algorithm falling into a local optimum, and thus, unreasonable planning occurs, the feasible solution of the fish swarm is used to replace the optimal solution, meaning that the fish swarm path point of the target point is recorded. Among these feasible solutions, the shortest path is selected as the optimal solution, and it is used as the final route of path planning.

**Figure 5:**
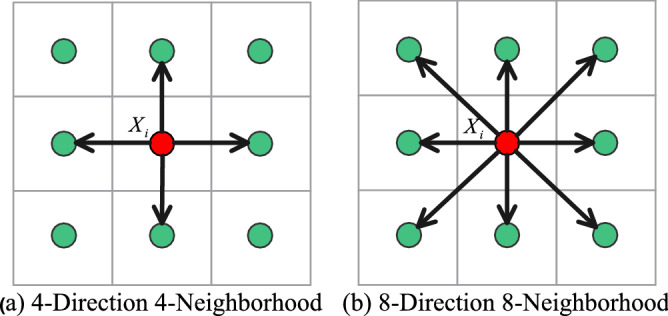
Step size range of the traditional fish school algorithm.

**Figure 6:**
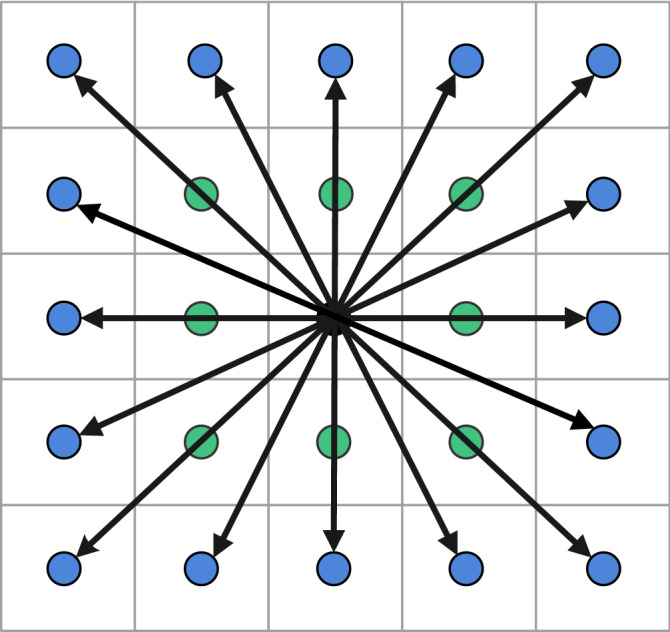
The step size range of the 16-Direction 24-Neighborhood.

In the grid map, the step size range $[1,\sqrt 2 ]$ of the traditional artificial fish swarm algorithm is usually 4-Direction 4-Neighborhood in Fig. 5a or 8-Direction 8-Neighborhood in Fig. 5b. However, to quickly reach the target position, it is necessary to increase the step size range. In the moving mode of 16-Direction 24-Neighborhood in this paper, the step size range of artificial fish is $[1,{2}\sqrt 2 ]$, as shown in Fig. 6.

**Figure 7:**
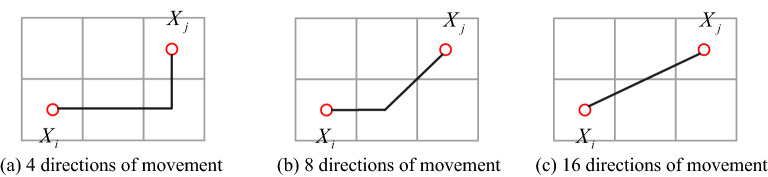
The planned path of a non-synchronized long range.

Compared with the traditional step size range, the proposed step size range has more direction selection and a wider step size range, which is beneficial for artificial fish to quickly reach the target point. In the following, an example is used to illustrate the advantages of the proposed 16-Direction 24-Neighborhood contrast to the traditional step size range. As shown in Fig. 7, the existing fish swarm should reach *X*_*j*_ from *X*_*i*_. Figure 7a, b are the paths planned by the traditional step size range. Figure 7c shows the planning path of the 16-Direction 24-Neighborhood. If the length of a grid is expressed as 1, the iteration times and path lengths of different step sizes can be calculated, as shown in Table 1. Table 1 shows that the step size range of the 16-Direction 24-Neighborhood proposed in this paper can find shorter paths with fewer iterations.

**Table 1:** Asynchronous long range result comparison.

Step range	Path length	Number of iterations
4-Direction 4-Neighborhood	3.00	3
8-Direction 8-Neighborhood	2.41	2
16-Direction 24-Neighborhood	2.24	1

**Figure 8:**
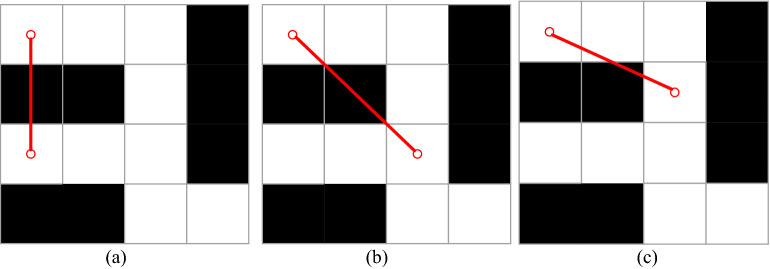
Irrational planning when the step range increases.

When the step range increases, obstacles may appear in the range, and fish can "surmount" the obstacles to reach the target point. However, for 2-D planar mobile robots, obstacle crossing must not occur. For mobile robots, the step size range proposed in this paper may lead to obstacle crossing, as shown in Fig. 8. The reason for this situation is that the traditional fish swarm algorithm determines the step size range through the Euclidean distance. However, with the increase in the step size range, the Euclidean distance cannot determine whether there is an obstacle between the two positions, and thus, obstacle crossing behavior occurs. The Dijkstra algorithm is introduced to confirm the step between two points as follows (7): If the equation is valid, there is no obstacle between the two points. Otherwise, the point does not satisfy the step range condition and is deleted from the neighborhood. However, when encountering the case in Fig. 8c, the Dijkstra algorithm is unable to identify the obstacle. Thus, formula (8) is introduced to judge it

7 $$\left\| {X_{i} - X_{j} } \right\| = {\text{Dijkstra}}(Dmap,X_{i} ,X_{j} )$$

8 $${\text{Allow\_area\_i}}() \cap {\text{Allow\_area\_j}}() = 2$$

${\text{Allow\_area\_i}}()$ represents the feasible region where the step size range of the current position *X*_*i*_ is $\left[ {1,\sqrt 2 } \right]$, and ${\text{Allow\_area\_j()}}$ is the feasible region where the random step size range of the neighborhood is $\left[ {1,\sqrt 2 } \right]$. Dijkstra() is the Dijkstra algorithm and *Dmap* is the adjacency matrix of the grid map. In the traditional algorithm, the probability of each attempt in each neighborhood is the same, thereby reducing the probability of a successful attempt. Therefore, the culled factor is introduced, and the neighborhood that has been culled will no longer be tried in the next attempt.

**Figure 9:**
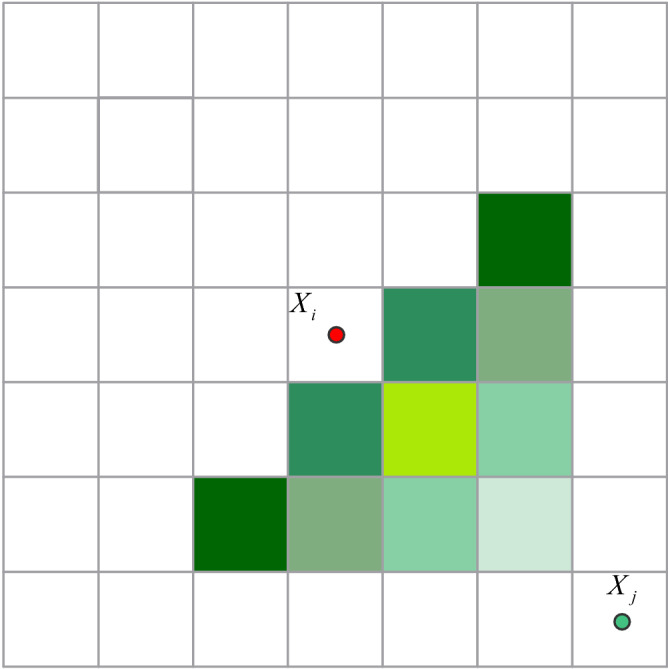
The range where artificial fish can try swim.

The artificial fish wants to reach *X*_*j*_ in the field of view, but due to the randomness of the artificial fish's swimming direction, it is necessary to determine whether the distance between the points in the neighborhood and *X*_*j*_ is less than the distance between *X*_*i*_ and *X*_*j*_, as shown in Fig. 9 below, and it is satisfied The neighborhoods of the above conditions are all filled with colors, and the remaining neighborhoods are not satisfied. In the traditional algorithm, the probability of each attempt in each neighborhood is the same, thereby reducing the probability of successful attempts. Therefore, the elimination factor is introduced, and the unsatisfactory elimination bit neighborhood will not be tried in the next attempt.

In the ASFA algorithm, the range of step size and field of vision are the main parameters affecting the convergence speed of the algorithm. The range of fields of vision has a very important influence on other behaviors of the fish swarm. The larger the range of the visual field, the more prominent the clustering behavior and following behavior of the fish swarm, which is beneficial for finding the global optimum. The small field of vision, fish foraging behavior and random behavior are more prominent, and a small range search will be more careful, but it will easily fall into a locally optimal solution. Therefore, the dynamic feedback field of vision is introduced to adjust the field of vision by feedback of the distance from the current position to the target point. Therefore, the fish can have a large field of vision in the early stage to quickly approach the target point. In the later stage, the fish can search carefully with a small field of vision and quickly reach the target point. The specific formula is as follows (9):

9 $$\left\{ {\begin{array}{*{20}l} {visual = visual\max *e^{{ - \lambda *\frac{t}{T}}} } \hfill \\ {\lambda = m*\left( {1 - \frac{dist}{{DistMin}}} \right)} \hfill \\ \end{array} } \right.$$

In the formula, *visual*_max_ is the maximum field of vision, *m* is the adjustable parameter, *t* is the current iteration number, *T* is the maximum iteration number, *dist* is the Euclidean distance from the target point in the fish swarm, *DistMin* is the Euclidean distance between the starting point and the endpoint, and *λ* is the feedback factor, which can be more effective in adjusting the field of vision.

The larger the step range, the faster the algorithm will converge, but when it reaches a certain range, the larger the step will oscillate, which will affect the convergence speed. Therefore, an adaptive step range is introduced. When the field of vision is less than a certain value, the step range-specific formulas (10–11) are as follows:

10 $$step = \alpha *step_{\min }$$

11 $$\alpha = \left\{ {\begin{array}{*{20}l} {2} \hfill & {\quad visual \ge step\max } \hfill \\ 1 \hfill & {\quad visual < step\max } \hfill \\ \end{array} } \right.$$

In the formula, *α* is the adjustment factor, *step*_min_ is the minimum step size range, and *step*_max_ is the maximum step size range.

**Figure 10:**
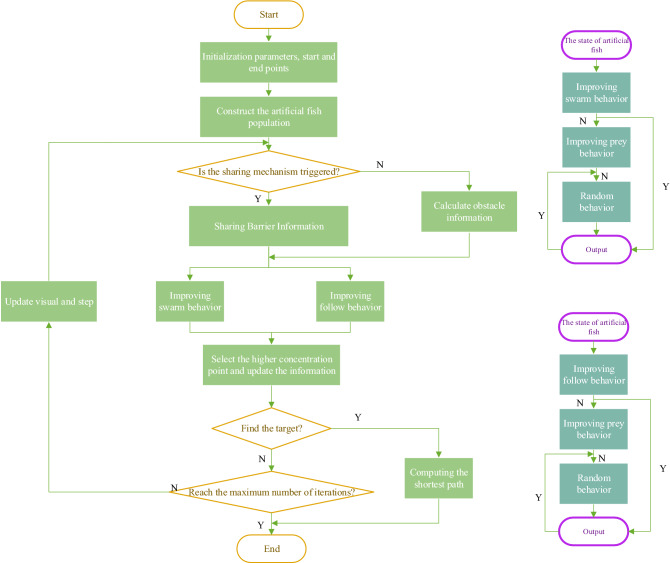
Flow chart of the improved artificial fish swarming algorithm.

The specific steps of the improved artificial fish swarm algorithm are shown in Fig. 10:

- *Step 1* We initialize the artificial fish parameters, such as the range of step length, range of vision, start point, end point, number of attempts, number of populations, and the crowding factor.
- *Step 2* We initialize the location of the fish population.
- *Step 3* We share the barrier information if the sharing mechanism is satisfied; otherwise, we calculate the barrier information.
- *Step 4* We perform preying behavior, swarming behavior, following behavior and random behavior and record the location information of the fish simultaneously.
- *Step 5* If the target point is found, we find the optimal solution among feasible solutions and end the loop; otherwise, we execute the next step.
- *Step 6* If the maximum number of cycles is reached, we end the cycle; otherwise, we return to step 3.

### Path smoothing

Since the path planning in this paper was performed under the grid map, whose initial path is composed of many folded line segments, there are places with sharp turns, where curvature and path orientation discontinuity occurs. At these turning points, the continuity of curvature and path orientation must be satisfied. The relationship between the radius of the rotation and curvature is as follows (12):

12 $$\kappa = \frac{1}{R} = \frac{{\left| {\dot{P}(t) \times \ddot{P}(t)} \right|}}{{\left| {\dot{P}(t)} \right|^{3} }}$$

In this paper, the Bezier curve is used to smooth the path. In References ^19,20^, the n-order Bezier curve of *n* + 1 control points $(P0,P1, \ldots ,Pn)$ is defined as follows:

13 $$P(s) = \sum\limits_{i = 0}^{n} {PiBn,s(s)} ,\quad Bn,i(s) = \left( \begin{gathered} n \hfill \\ i \hfill \\ \end{gathered} \right)s^{i} (1 - s)^{n - i}$$

In formula (17), *s* is $0 \le s \le 1$, and $Bn,i(s)$ are Bernstein polynomials.

In this paper, the third-order Bezier curve is used to smooth the path; each of the four control points controls a curve. As shown in the above figure, *E*_0_, *B*_0_, *B*_1_, *B*_2_ and *E*_2_, *A*_0_, *A*_1_, *A*_2_ are the points passed by the planned paths *E*_0_*E*_1_ and *E*_1_*E*_2_. *E*_1_ is the inflection point of the path, *u*_1_, *u*_2_ is the unit vector of $\overrightarrow {{E_{{1}} E_{{0}} }}$ and $\overrightarrow {{E_{1} E_{2} }} ,C = (C_{x} ,C_{y} ) = \overrightarrow {{E_{0} E_{2} }}$ is the vector connecting *E*_0_ and *E*_2_, *α* and *β* are the angles of $\overrightarrow {{B_{{2}} A_{{2}} }}$ and $\overrightarrow {{B_{2} E_{1} }} ,\overrightarrow {{E_{0} E_{1} }}$ and $\overrightarrow {{E_{{1}} E_{{2}} }}$.

According to the smoothing theory of continuous third-order Bezier curves, the third-order Bezier curve segments satisfy formula (14) as follows:

14 $$\begin{aligned} F\left( \alpha \right) & = \cos^{2} (\beta - \alpha )\sin \alpha [(c_{1} + \, 6\cos^{2} \alpha )Dy \\ & \quad - 6C_{x} \cos \alpha \sin \alpha ] + \, \cos^{2} \alpha \, \sin (\beta - \alpha )[c_{1} (C_{y} \cos \beta \\ & \quad - C_{x} \sin \beta ) + 6\cos \, (\beta - \alpha )(C_{y} \cos \alpha - C_{x} \sin \alpha )] = 0 \\ \end{aligned}$$

The above curve is composed of two Bezier curves and is determined by eight control points. The first curve is controlled by four points as follows:

15 $$\left\{ {\begin{array}{*{20}l} {B_{{0}} = E_{{1}} + d_{{1}} u_{{1}} } \hfill \\ {B_{{1}} = B_{{0}} - p_{b} u1} \hfill \\ {B_{{2}} = B_{{1}} - q_{b} u_{{1}} } \hfill \\ {B_{{3}} = B_{{2}} - f_{b} u_{d} } \hfill \\ \end{array} } \right.$$

The second curve is controlled by four points:

16 $$\left\{ {\begin{array}{*{20}l} {A_{0} = E_{1} + d_{2} u_{2} } \hfill \\ {A_{{1}} = A_{0} - p_{a} u_{2} } \hfill \\ {A_{{2}} = A_{{1}} - q_{a} u_{2} } \hfill \\ {A_{{3}} = A_{{2}} - f_{a} u_{d} } \hfill \\ \end{array} } \right.$$

In Eqs. (15) and (16) above, *d*_1_ and *d*_2_ are the lengths of *E*_1_*B*_0_ and *E*_1_*A*_0_ respectively, and *u*_d_ is the unit vector of $\overrightarrow {{B_{2} A_{2} }}$. Other parameters can be expressed by Eqs. (17.1–17.4) as follows:

17.1 $$\left\{ {\begin{array}{*{20}l} {p_{a} = c_{2} q_{a} } \hfill \\ {p_{b} = c_{2} q_{b} } \hfill \\ \end{array} } \right.$$

17.2 $$\left\{ {\begin{array}{*{20}l} {q_{a} = \frac{{q_{b} \cos^{2} \alpha \sin (\beta - \alpha )}}{{\sin \alpha \cos^{2} (\beta - \alpha )}}} \hfill \\ {q_{b} = \frac{{(c_{2} + 4)C_{y} {\text{cos}}^{{2}} (\beta - \alpha )sin\alpha }}{{[(c_{1} - 6)\cos \alpha \sin (\beta - \alpha ) + 6\sin \beta ]\cos \alpha \sin \beta }}} \hfill \\ \end{array} } \right.$$

17.3 $$\left\{ {\begin{array}{*{20}l} {f_{a} = \frac{6}{{c_{2} + 4}}q_{a} \cos (\beta - \alpha )} \hfill \\ {f_{b} = \frac{6}{{c_{2} + 4}}q_{b} \cos \alpha } \hfill \\ \end{array} } \right.$$

17.4 $$\left\{ {\begin{array}{*{20}l} {c_{1} = 7.2364} \hfill \\ {c_{2} = \frac{2}{5}(\sqrt 6 - 1)} \hfill \\ \end{array} } \right.$$

**Figure 11:**
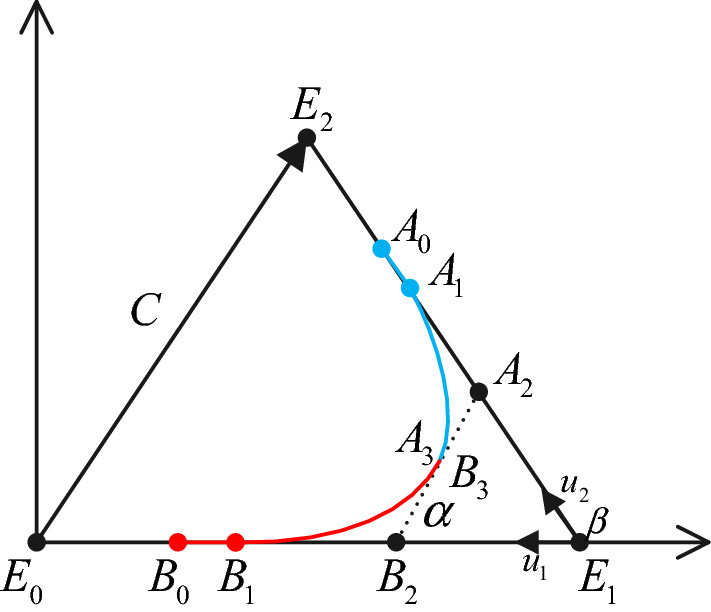
The Bezier curve model.

To reduce the number of parameters, the mirror law is introduced so that *d* = *d*_1_ = *d*_2_, *α* = *β*/2; then from Fig. 11, we obtain the Eq. (18) as follows:

18 $$\left\{ {\begin{array}{*{20}l} {C_{x} = l + l\cos \beta } \hfill \\ {C_{y} = d\sin \beta } \hfill \\ \end{array} } \right.$$

Substituting (18) into (14) to simplify gives Eq. (19) as follows:

19 $$\begin{aligned} F\left( \alpha \right) & = c_{1} d\sin \beta [cos^{2} (\beta - \alpha )\sin \alpha - \cos^{2} \alpha \sin (\beta - \alpha )] \\ & \quad + 6d\cos^{2} (\beta - \alpha )\sin \alpha \left[ {\cos^{2} \alpha \sin \beta - \cos^{2} \frac{\beta }{2}\sin 2\alpha } \right] \\ & \quad + 6d\cos^{2} \alpha \sin (\beta - \alpha )\cos (\beta - \alpha )[\sin (\beta - \alpha ) - \sin \alpha ] \\ \end{aligned}$$

Substituting (18) into *q*_*a*_, *q*_*b*_, the Eqs. (20.1–20.3) obtained by simplifying as follows:

20.1 $$q_{b} = \frac{{(c_{2} + 4))d\sin 2\alpha \cos^{2} \alpha \sin \alpha }}{{[(c_{1} - 6)\cos \alpha \sin \alpha + 6\sin 2\alpha ]\cos \alpha \sin 2\alpha }} = c_{3} d$$

20.2 $$q_{a} = \frac{{q_{b} \cos^{2} \alpha \sin \alpha }}{{\sin \alpha \cos^{2} \alpha }} = q_{b}$$

20.3 $$c_{3} = \frac{{c_{2} + 4}}{{c_{1} + 6}}$$

Thus, simplifying Eqs. (17.1–17.4) yields the Eqs. (21.1–21.3) as follows:

21.1 $$q_{a} = q_{b} = c_{3} d$$

21.2 $$p_{a} = p_{b} = c_{2} c_{3} d$$

21.3 $$f_{a} = f_{b} = \frac{{6c_{3} \cos \alpha }}{{c_{2} + 4}}d$$

In Formula (21) above, as long as the size is determined, eight control points can be determined, and the third-order Bezier curve can be constructed. In general, the first line of the two adjacent straight lines is used to construct an arc satisfying the maximum rotation curvature. At the last point A of the first arc, the first and second derivatives of the third-order Bezier curve are as follows (22):

22 $$\left\{ {\begin{array}{*{20}l} {\dot{P}(1) = 3\overrightarrow {{f_{b} }} } \hfill \\ {\ddot{P}(1) = 6\overrightarrow {{f_{b} }} - 6\overrightarrow {{q_{b} }} } \hfill \\ \end{array} } \right.$$

By substituting formula (22) into (12), we can obtain the equation as follows:

23 $$\begin{aligned} \kappa_{\max } & = \frac{{\left| {3\overrightarrow {{f_{b} }} \times (6\overrightarrow {{f_{b} }} - 6\overrightarrow {{q_{b} }} )} \right|}}{{\left| {3\overrightarrow {{f_{b} }} } \right|^{3} }} = \frac{{2h_{b} \sin \alpha }}{{3f_{b}^{2} }} \\ & = \left( {\frac{{\left( {c_{2} + 4} \right)^{3} }}{{54c_{3} }}} \right)\frac{\sin \alpha }{{d\cos^{2} \alpha }} \\ \end{aligned}$$

Therefore when *d* ≠ 0 or *α* ≠ 90°, the *κ*_max_ of the third-order Bezier curve is continuous.

Because the planned path has multipoint collinearity, this situation does not meet the continuity condition (*α* ≠ 90°) of the above three-order Bezier curve, so the intermediate redundant points are removed. In this paper, the rank method of matrix is used to determine whether the three points (*x*_1_, *y*_1_), (*x*_2_, *y*_2_) and (*x*_3_, *y*_3_) are linearly correlated as follows:

24 $$a = \left( {\begin{array}{*{20}c} {x_{1} } & {y_{1} } & 1 \\ {x_{2} } & {y_{2} } & 1 \\ {x_{3} } & {y_{3} } & 1 \\ \end{array} } \right)$$

**Figure 12:**
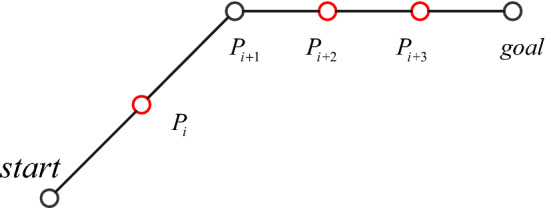
Collinear optimization of the path points.

In Eq. (24), if a is a full rank matrix, then the three points are not collinear, then (*x*_2_, *y*_2_) is reserved, otherwise (*x*_2_, *y*_2_) is discarded. In the algorithm, collinear optimization is carried out for the planned path. First, the number of planned path points *i* is calculated, and the starting point is put into *Final*. Then, three points $P{}_{j - 1},P_{j} ,P_{j + 1} (j = 2,3 \ldots ,i - 1)$ are removed to judge whether they are collinear. If they are collinear, *P*_*j*_ is put into *Final*, otherwise *P*_*j*_ is discarded until the end of the *j* + 1 > *i* cycle, and finally the target point is put into *Final*. As shown in Fig. 12, the improved artificial fish swarm algorithm shows that the path points are $(start,P_{i} ,P_{i + 1} ,P_{i + 2} ,P_{i + 3} ,goal)$ and that the path points after the path passing collinear optimization are $(start,P_{i + 1} ,goal)$, which satisfies the conditions of continuous third order Bezier curve.

## Results

To verify the effectiveness and feasibility of the combination of the improved artificial fish swarm algorithm and the continuous Bessel curve smoothing algorithm proposed in this paper, comparative simulation experiments are carried out in grid environments with different complexities.

### Comparison of artificial fish school algorithms

**Figure 13:**
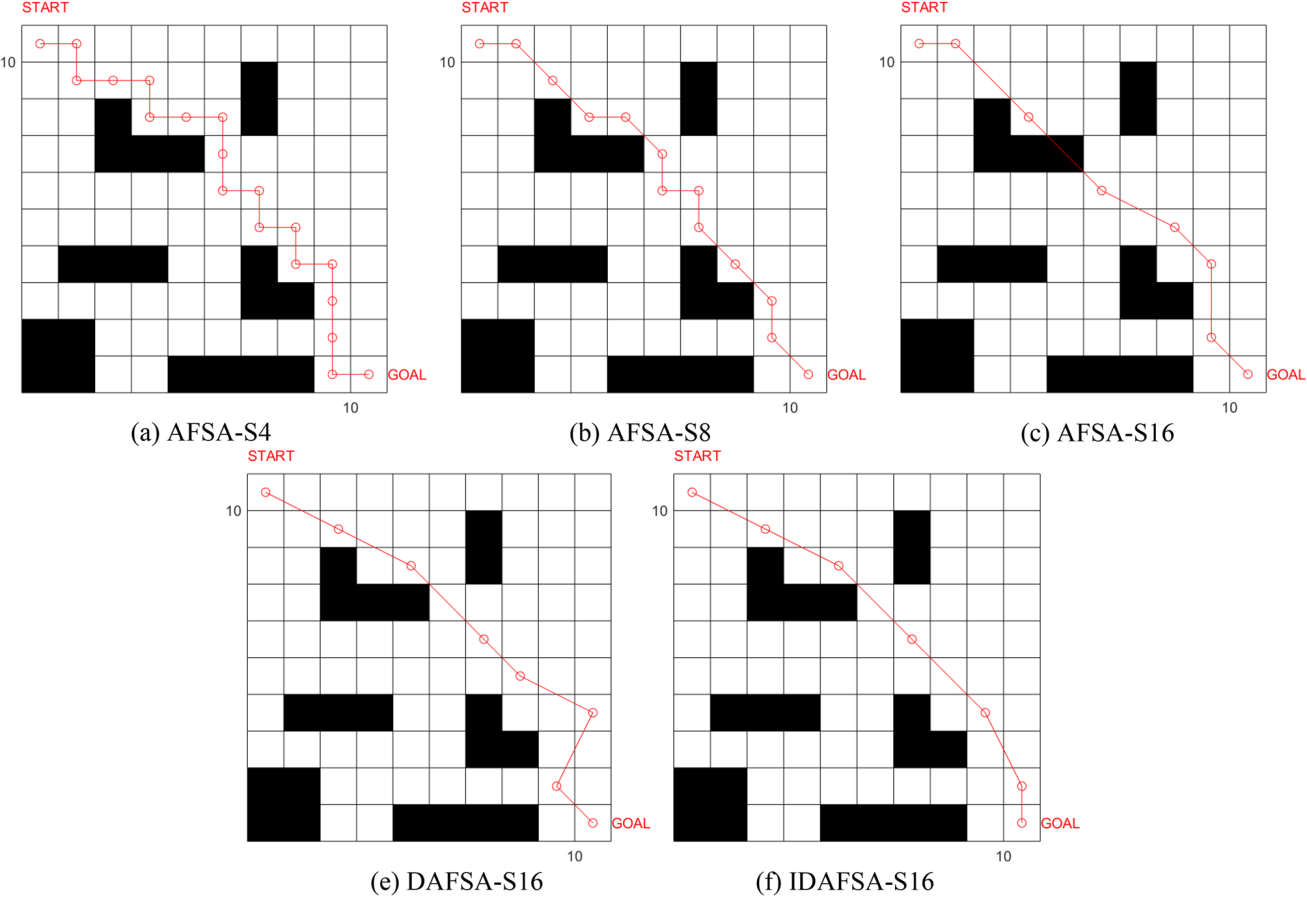
Comparison of simulation plots of paths planned by different algorithms in a 10 × 10 grid environment.

In a 10 × 10 grid map environment, several comparison experiments are carried out, and the basic parameters of the algorithm are the same, namely *N* = 50, *try-number* = 8, *MAXGEN* = 100, *visual* = 5, and $\partial = 0.618$. However, the swimming modes are 4-Direction 4-Neighborhood (AFSA-S4) and 8-Direction 8-Neighborhood (AFSA-S8), 16-Direction 24-Neighborhood (AFSA-S16), Dijkstra-based 16-Direction 24-Neighborhood (DASFA-S16), and the improved DAFSA-S16 algorithm (IDFSA-S16) is proposed in this paper. The basic parameters of IDAFSA-S16 are *visual*_max_ = 5, $step_{\max } = 2\sqrt 2 ,step_{\min } = \sqrt 2$, and *m* = 17. In a 10 × 10 grid environment, the paths planned by different algorithms are shown in Fig. 13.

**Figure 14:**
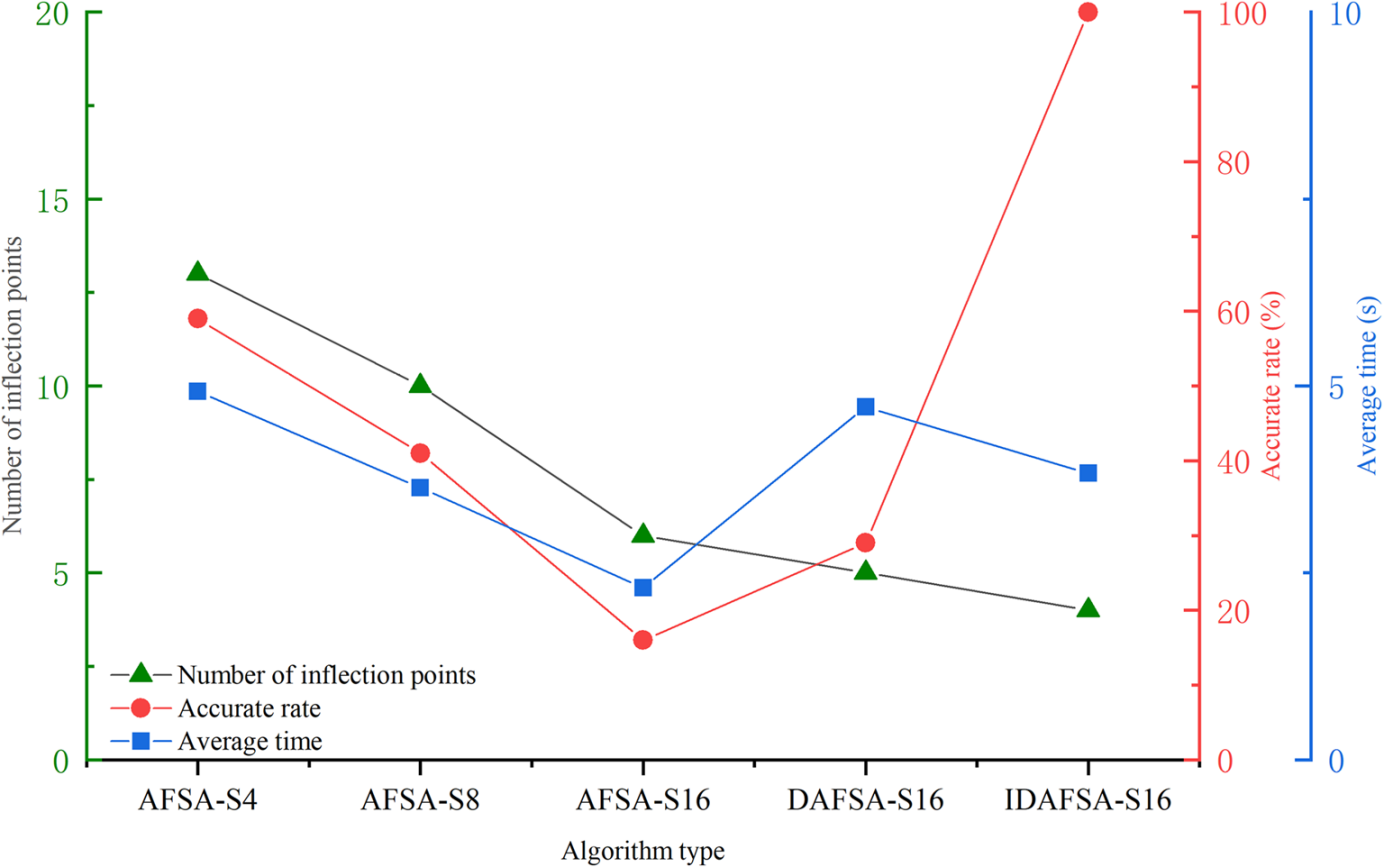
Comparison of the number of inflection points, average time and planning accuracy of different algorithms in a 10 × 10 grid environment.

**Figure 15:**
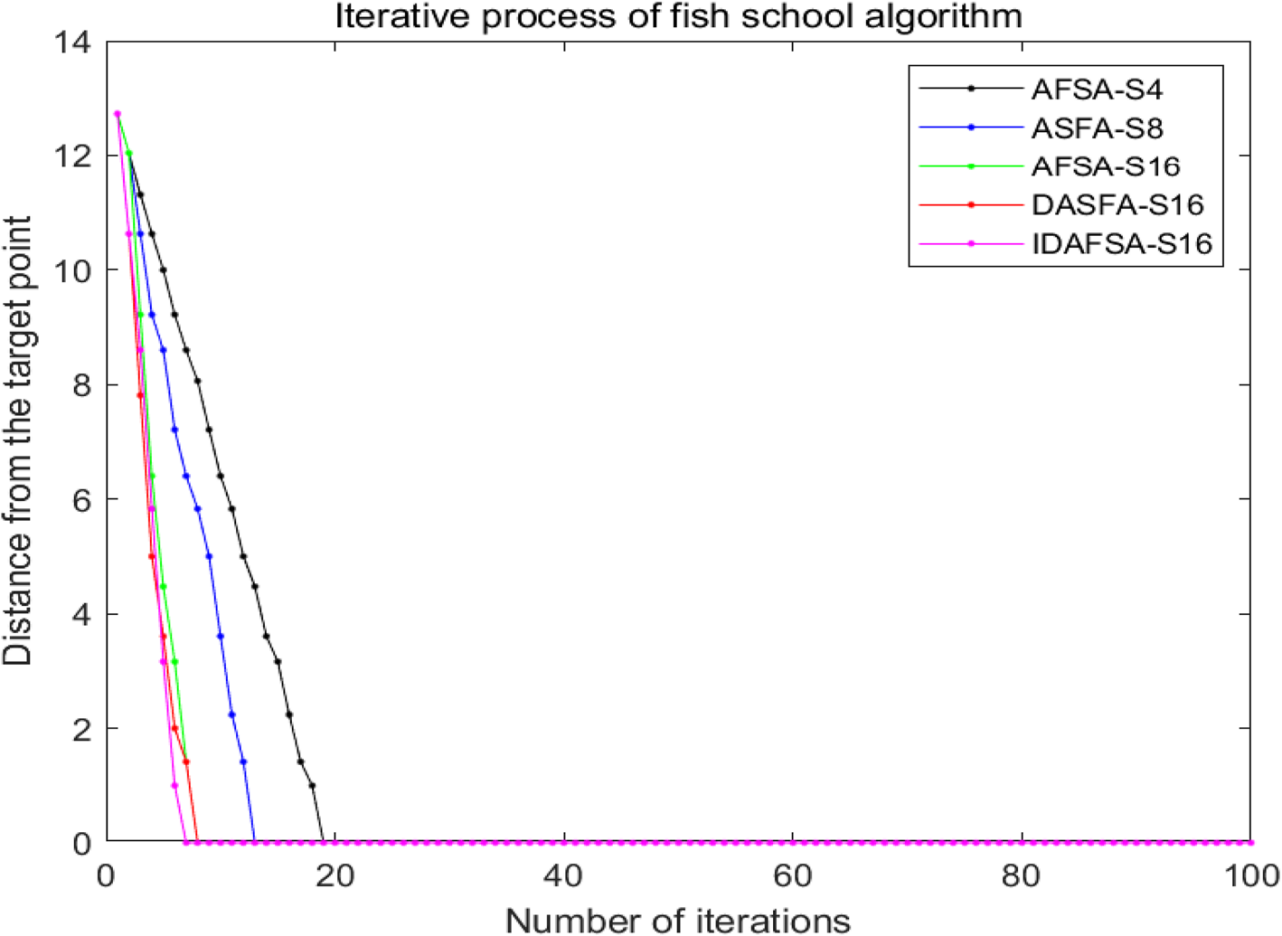
Comparison of the number of iterations of different algorithms in a 10 × 10 grid environment.

From the analysis of Table 2, Figs. 13, 14 and 15, the path planned by ASFA-S4 is too cumbersome, with too many iterations and too much calculation. The average planning path length is relatively large and there are many inflection points; the average planned path length of ASFA-S8 is reduced by 16.5% compared with that of AFSA-S4, but the shortest and longest paths fluctuate greatly and unstable, with relatively many inflection points. The average planning path length of AFSA-S16 is 23.6% and 8.4% less than that of AFSA-S4 and AFSA-S8, respectively, with relatively fewer inflection points and the least number of operations, but it is not stable and prone to unreasonable planning, and global solution oscillation occurs due to the large range of the later field of view, which affects the algorithm's later optimization search.DAFSA-S16 introduces Dijkstra algorithm on the basis of AFSA-S16. DAFSA-S16 introduces the Dijkstra algorithm on the basis of AFSA-S16 to further confirm the obstacle information within the step length, and the simulation results show that the accuracy of the algorithm is improved. At the same time, the average path length of DAFSA-S16 is reduced by 24.3%, 9.3%, and 1.0% compared with AFSA-S4, AFSA-S8, and AFSA-S16, respectively, and the inflection point is also relatively small. Since the large field of vision in the late stage will affect the optimization of the algorithm, the late oscillation of the algorithm shown in Fig. 13d above appears. IDSFA-S16 can effectively solve the behavior of unreasonable crossing of the planning path, while the feedback field of view range can effectively solve the problem of global solution oscillation at the later stage of the algorithm. The average path length is 27.4%, 13%, 5.0%, 4.0% less than the above algorithm, and the accuracy of the algorithm planning can also reach 100%, while the inflection point is relatively less, and the path is smoother, but the amount of operations is relatively increased.

**Table 2:** Comparison of path lengths of different algorithms in a 10 × 10 grid environment.

Algorithm	Longest path	Shortest path	Average
AFSA-S4	18.0000	20.6051	18.6758
ASFA-S8	17.7568	14.8284	15.5852
ASFA-S16	16.4721	13.7726	14.2722
DASFA-S16	15.0711	13.7726	14.1289
IDAFSA-S16	14.1289	13.1869	13.5567

**Figure 16:**
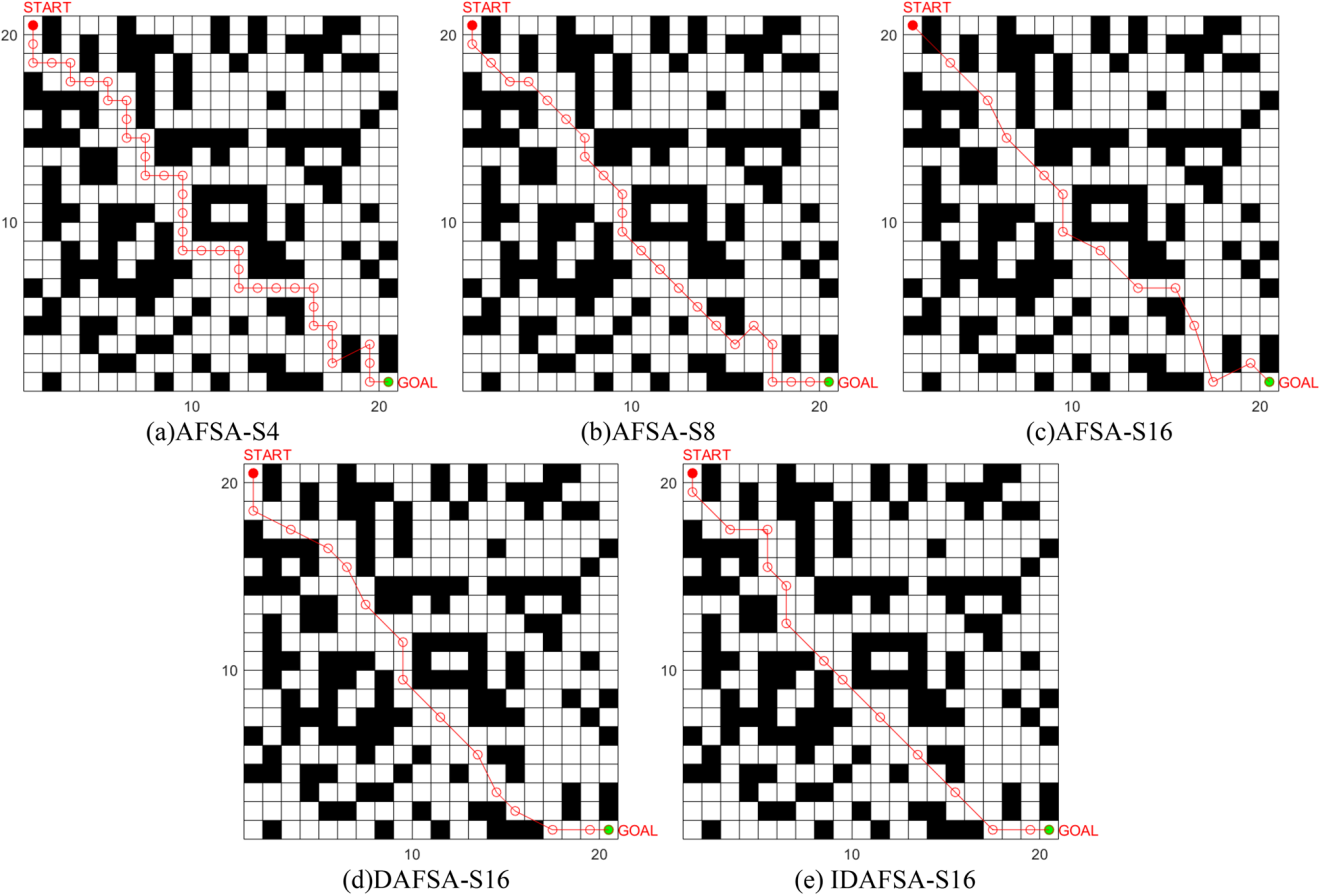
Comparison of simulation plots of paths planned by different algorithms in a 20 × 20 grid environment.

**Figure 17:**
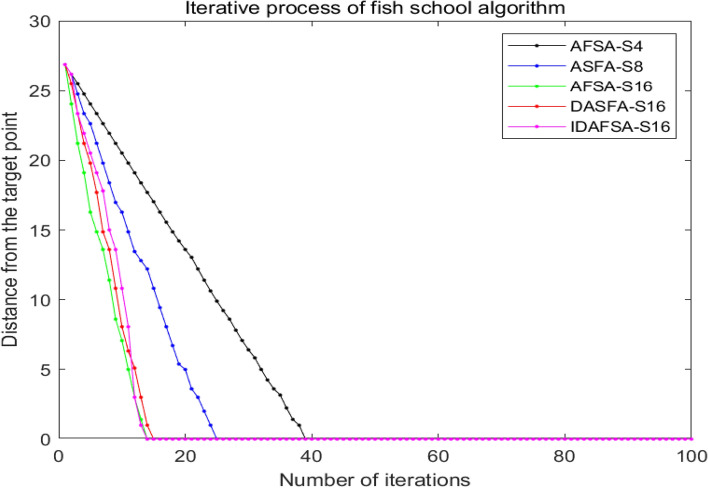
Comparison of the number of iterations of different algorithms in a 20 × 20 grid environment.

In a 20 × 20 grid map environment, several comparison experiments are carried out, and the basic parameters of the algorithm are the same, namely, *N* = 50, *try-number* = 8, *MAXGEN* = 100, *visual* = 5, and $\partial = 0.618$. However, the swimming modes are 4-Direction 4-Neighborhood (AFSA-S4) and 8-Direction 8-Neighborhood (AFSA-S8), 16-Direction 24-Neighborhood (AFSA-S16), Dijkstra-based 16-Direction 24-Neighborhood (DASFA-S16), and the improved DAFSA-S16 algorithm (IDFSA-S16) proposed in this paper. The basic parameters of IDAFSA-S16 are *visual*_max_ = 5, $step_{\max } = 2\sqrt 2 ,step_{\min } = \sqrt 2$, and *m* = 10. Table 3 shows the comparison of planned path lengths in a 20 × 20 grid environment, Fig. 16 shows the planning path under the grid environment of 20 × 20, and Fig. 17 shows the comparison iteration diagram.

**Table 3:** Comparison of planned path lengths in a 20 × 20 grid environment.

Algorithm	Longest path	Shortest path	Average
AFSA-S4	43.2366	39.2361	39.5647
ASFA-S8	31.2132	29.2132	30.6538
ASFA-S16	34.9494	27.9367	29.6522
DASFA-S16	30.9596	28.7814	29.5581
IDAFSA-S16	30.4361	29.2648	29.4916

**Figure 18:**
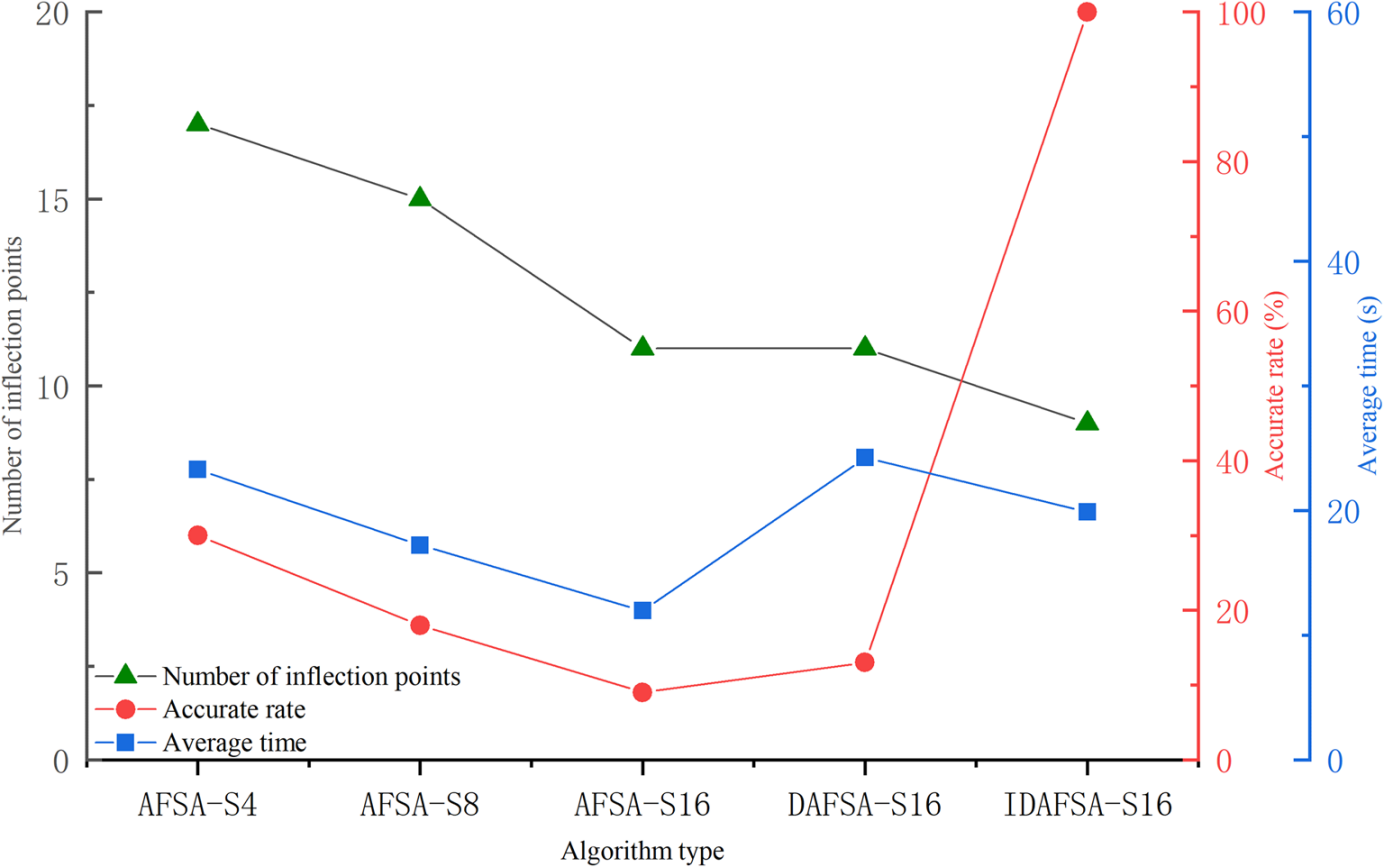
Comparison of the number of inflection points, average time and accuracy of different algorithms in a 20 × 20 grid environment.

The analysis of Table 3, Figs. 16, 17 and 18 shows that in the relatively complex environment, the path planned by AFSA-S4 is very complex, with the largest number of inflection points and iterations, as well as the largest number of algorithm operations. There is also an unreasonable cross-barrier phenomenon. The difference between the longest path and the shortest path planned by ASFA-S8 is large, and the global optimal solution and the local optimal solution interfere with each other, leading to unreasonable planning paths. AFSA-S16 is difficult to plan the route correctly. DAFSA-S16 planning accuracy is improved, and the inflection point is relatively small, but it is also difficult to control the local optimum and the global optimum interfering with each other. By replacing the optimal solution with a feasible solution, IDAFSA-S16 can ensure rationality and stability under the planned path, while also reducing the number of inflection points and the number of iterations. Then, the algorithm operations are relatively reduced, and the average path lengths are 25.4%, 3.9%, 0.5% and 0.2% less than those of the above algorithms.

### Path Smoothing

**Figure 19:**
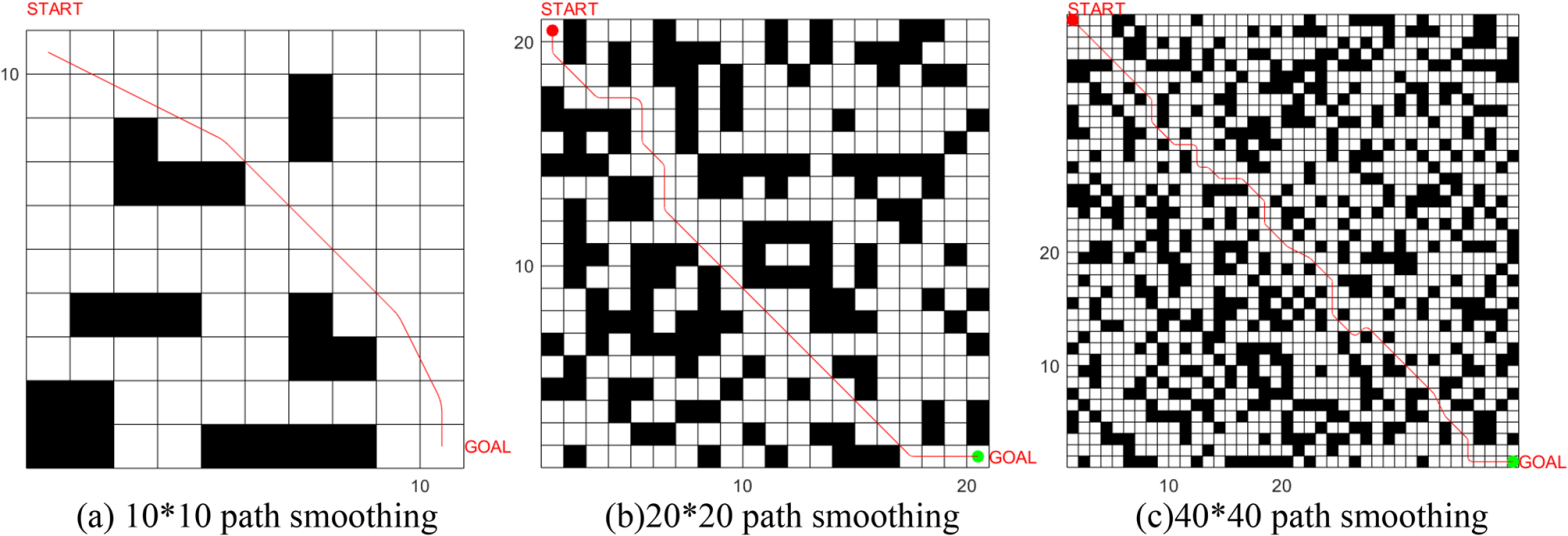
Simulation of path smoothing in different raster environments.

To verify the effectiveness of the path smoothing of the third-order Bessel curve and the continuity after collinear optimization, simulations are carried out in grid environments with different complexities, as shown in Fig. 19. From the simulation results, it can be seen that in different environments, by introducing the third-order Bessel curve, the path is smoothed so that the original sharp inflection point becomes smooth, and the actual control meets the kinematic and dynamic characteristics of the mobile robot. By effectively avoiding obstacles and satisfying feasibility, the continuity of the rotation angle and path curvature of the mobile robot is realized.

## Discussion and conclusion

Aiming at robot path planning, this paper proposed a method combining third-order Bessel curves with an improved artificial fish swarm algorithm. First, morphological processing, such as the expansion of obstacles, was carried out, and during the path planning process, an artificial fish swimming range of 16 directions and 24 fields based on Dijkstra's algorithm was introduced to provide the movement rules of the artificial fish, which improves the accuracy of planning while reducing the inflection points. At the same time, by adding the sharing mechanism, the number of operations of the fusion algorithm was reduced. By introducing the feasible solution, the problem of mutual interference between the local optimal solution and the global optimal solution was solved. By introducing the feedback field of view range, the oscillation phenomenon caused by an excessively large field of view in the later stage of the algorithm was avoided. The improved artificial fish swarm algorithm proposed in this paper generates a collision-free, inflection-point less and shorter path connected by a sequential sequence of path points. Finally, smoothing by using the theory of cubic Bessel curves ensures the continuity of direction and curvature of any point on its path, while made it satisfy the minimum rotation radius to reduce the mechanical structure damage of the robot. By comparing the simulation results of the proposed algorithm with the traditional fish swarm algorithm, it can be seen that the proposed fusion algorithm had the shortest average path, the least number of inflection points, and was more consistent with the kinematic characteristics of the robot, while also ensured a 100% correct planning rate. At the same time, the proposed fusion algorithm introduces Dijkstra's algorithm, which causes an increase in computation, but then the fish sharing mechanism was introduced to offset part of the computation.

In summary, it is sufficient to prove the effectiveness and superiority of the proposed algorithm in this paper. However, the proposed algorithm was only tested in a raster environment and not in a real environment. In future work, we will conduct experiments and debugging in a real environment. We are also interested in using IDAFSA in multirobot collaborative path planning.
